# Reduced Self-Perception of Fatigue after Intake of *Panax ginseng* Root Extract (G115^®^) Formulated with Vitamins and Minerals—An Open-Label Study

**DOI:** 10.3390/ijerph18126257

**Published:** 2021-06-09

**Authors:** Anne-Laure Tardy, Beatrice Bois De Fer, Salvador Cañigueral, David Kennedy, Andrew Scholey, Simon Hitier, Alexia Aran, Etienne Pouteau

**Affiliations:** 1 Sanofi Global Consumer Health Care, 94250 Gentilly, France; Beatrice.Bois-de-Fer@sanofi.com (B.B.D.F.); Simon.Hitier@sanofi.com (S.H.); alexia.aran@sanofi.com (A.A.); Etienne.Pouteau@sanofi.com (E.P.); 2 Unit of Pharmacology, Pharmacognosy and Therapeutics, Faculty of Pharmacy and Food Sciences, University of Barcelona, 08007 Barcelona, Spain; s.canigueral@ub.edu; 3 Brain, Performance and Nutrition Research Centre, Faculty of Health and Life Sciences, Northumbria University, Newcastle-upon-Tyne NE1 8ST, UK; david.kennedy@northumbria.ac.uk; 4 Centre for Human Psychopharmacology, Swinburne University, Melbourne, VIC 3122, Australia; andrew@scholeylab.com

**Keywords:** *Panax ginseng* G115^®^, fatigue, energy, vitamins, minerals

## Abstract

Background: Unexplained fatigue is a common complaint. When underlying disease causes have been eliminated, lifestyle measures and supplementation can be indicated. Elaborating on clinical findings that G115^®^, a dry extract from the root of *Panax ginseng,* combined with vitamins and minerals could alleviate fatigue, this open label study aimed at assessing its effect on perceived fatigue and energy. Methods: Healthy adults self-reporting fatigue (*n* = 103) completed the Multidimensional Fatigue Inventory questionnaire. They rated their perceptions of mental and physical fatigue, energy, performance, and stress at baseline and 15, 30, 60 and 90 days after a daily intake of 40 mg G115^®^ formulated with vitamins and minerals. Results: Compared with baseline values, mean self-perception of general fatigue was reduced by −7.55 units [95% CI: −8.44; −6.66] (−41.8%, *p* < 0.0001) at 90 days. All assessed perception ratings (mental and physical fatigue, reduced activity and motivation, performance, and stress) were significantly and steadily improved from two weeks after supplementation up to study’s end. Overall satisfaction with the ability of the product to reduce fatigue reached 85% at Day 90. Conclusion: Daily intake with G115^®^ extract formulated with vitamins and minerals suggests an improvement of self-perception of fatigue and energy in a fatigued adult population.

## 1. Introduction

The concept of energy, in everyday language, is associated with the feelings of vitality and well-being that make our daily physical or intellectual activities possible. Conversely, fatigue is usually described as a perceived lack of energy [1]. Energy and fatigue are thus interrelated opposite perceptions, each characterized by physical and mental components. In healthy individuals, the main causes of fatigue and lack of energy are related to professional occupation, factors related to domestic and social events, and individual lifestyle or behavior [2,3]. Beyond perceptions and feelings, fatigue and lack of energy also result in decreased physical, cognitive and psychological performance [4]. Recommendations and the means to alleviate fatigue and its consequences are frequently requested by physicians [5], who usually promote a healthy lifestyle, including a balanced diet and sufficient sleep, having eliminated any underlying, disease-associated cause [6]. Alleviation of fatigue and the maintenance of general health have been reported as the most common motivations for taking supplements [7].

Vitamins and minerals are of key importance for their contribution to normal energy-yielding metabolism in the body. They are essential for various metabolic reactions that enable the transformation of carbohydrates, lipids, and proteins from food into chemical energy. In addition to this energy-yielding function, vitamins and minerals have critical functions in pathways that support the synthesis of nucleic acids, the transport of oxygen, and neuronal function, thereby impacting on muscular, cognitive and psychological function, as well as on mental and physical fatigue [4,8]. Several observational studies have reported that fatigue and poorer functioning are associated with lower status in some micronutrients, such as iron [9] or vitamin C [10].

The root of *Panax ginseng* C.A. Meyer, also known as Asian or Korean ginseng, has been used for centuries in Chinese and other traditional Asian medicines, for a wide variety of ailments, including, but not restricted to, general weakness, lack of appetite, and anxiety. Possible underlying mechanisms include antioxidant properties, regulation of carbohydrate metabolism, promotion of the mitochondrial function, neuroprotection and the prevention of neurotransmitter disorders in the central nervous system, according to a recent systematic review [11]. It has been used in the Western world since the end of the 19th century, as a traditional medicine indicated in cases of tiredness, weakness and decreased mental and physical capacity [12,13,14].The roots of *Panax ginseng* are authorized as a herbal extract for use within food supplements in many countries, such as in France [15], Belgium, [16] or Italy [17]. A specific extract referred to as G115^®^, standardized to contain 4% of ginsenosides, considered the active components of the herbal drug was first investigated in the early 1980s. In addition to preclinical studies in animals or in vitro, aimed at identifying potential mechanisms of action of *Panax ginseng* [18], clinical studies have documented the physiological effects of G115^®^ over the last 40 years [19,20]. When focusing only on the clinical trials that address cognition, fatigue, or well-being outcomes, more than 20 studies have been undertaken up to now, most of them double blinded and placebo-controlled trials using G115^®^ alone or in combination with vitamins and minerals. Indeed, G115^®^ was demonstrated to help maintain mental performance, especially in case of fatigue and highly demanding tasks [21,22,23,24,25,26]. In addition, the combination of G115^®^ extract with vitamins and minerals was able to improve the quality of life in subjects experiencing high levels of physical and mental stress [27] or presenting an overall low quality of life [28]. This formulation also helped to reduce memory deficits and self-reported fatigue in shift-workers [29]. Thus, there is a consistent effect of Panax Ginseng G115^®^ extract, alone or in combination with vitamins and minerals, on memory, and especially on secondary memory, a key component of working memory. In addition, most studies conclude to an improvement in well-being and the quality of life and a reduction in perceived mental fatigue, which is particularly visible when subjects are in a challenging situation, such as for shift-workers, people with enhanced stress or poor quality of life at baseline, or when challenged by a demanding cognitive task.

Given the relationship between micronutrient status and fatigue, and the described effects of the root of *Panax ginseng*, a study investigating a marketed food supplement containing 11 vitamins, 6 minerals and G115^®^ *Panax ginseng* extract was set up in healthy, non-elderly adult participants reporting a subjective fatigue. The primary objective was to evaluate the self-perception of general fatigue after 3 months of daily intake with the combination of vitamins, minerals and G115^®^ *Panax ginseng* extract, and the secondary objectives were to evaluate the self-perception of physical fatigue, mental fatigue, reduced activity, motivation, and stress, and assess the satisfaction with the product usage. All these perceptions were evaluated at regular intervals during a 3-month period, in order to provide insights to the kinetics of expected changes.

## 2. Material and Methods

### 2.1. Ethics

This study was conducted from December 2018 to March 2019 in two centers in Spain (Madrid and Valencia), as a behavioral study test in healthy volunteers with a marketed food supplement and without invasive methods of evaluation. The study is compliant with the International Code on Market, Opinion and Social Research and Data Analytics [30]. It was approved by the Parc Taulí’s CEIm Ethics committee in Barcelona, which considers that the necessary requirements for the appropriateness of the protocol in relation to the objectives of the study are met and the foreseeable risks and discomfort to the subject are justified. All participants provided written informed consent prior to participation and received financial compensation.

### 2.2. Study Participants

Male and female subjects in good overall health and aged 18 to 45 were included if they declared a lack of energy and vitality, and persistent fatigue for at least two weeks. Inclusion was also dependent on the subject presenting a score ≥10 on the dimension of general fatigue of the Multidimensional Fatigue Inventory (MFI) and a score ≥3 for at least ten of the 20 questions of the MFI [31]. Subjects were excluded if they were working at night, taking any food supplements in the month before study onset, or if their usual daily intakes exceeded four cups of coffee, two glasses of alcohol, or five cigarettes. Weekly intakes exceeding three energy drinks or five snacks and performing more than 45-min medium to intense physical exercise more than twice weekly were also exclusion criteria.

A total of 130 subjects were screened, of whom 105 subjects were included. Baseline characteristics are described in Table 1, including scores on the MFI before the study started.

### 2.3. Study Product

The supplement consists in soft gelatin capsules, each containing 1.1 mg vitamin B1, 1.4 mg vitamin B2, 16 mg vitamin B3, 1.4 mg vitamin B6, 50 µg vitamin B8, 200 µg vitamin B9 (folate), 2.5 µg vitamin B12, 800 µg vitamin A, 60 mg vitamin C; 5 µg vitamin D, 12 mg vitamin E, 1 mg copper, 10.5 mg iron, 2 mg manganese, 55 µg selenium, 1.5 mg zinc and 120 mg calcium. The recommended daily dose is one capsule which delivers amounts within the order of magnitude of the recommended daily dose for an adult for each vitamin and mineral described. In addition, each capsule contains 40 mg of G115^®^, which is a dry extract obtained from the root of *Panax ginseng* (C.A. Meyer) (drug-extract ratio 3–7:1), using ethanol 40% *v/v* as extraction solvent. G115^®^ is standardized to 4% of ginsenosides (sum of ginsenosides Re, Rf, Rg1, Rg2, Rb1, Rb2, Rc and Rd) by the addition of excipients (43–68% lactose, and 2% silicon dioxide). Quality assurance for all the manufacturing steps, standardization and quality control of the process guarantees the same batch-to-batch content and composition of ginsenosides, which is the basis for reproducibility of the safety and efficacy of the final product [32].

### 2.4. Study Procedure

Participants were asked to consume one capsule each morning for 90 days, and to evaluate their self-perception of fatigue using MFI and of stress using Numerical Rating Scales (NRS), as well as their overall satisfaction with the product, in the evening (before dinner) at baseline and after 1, 15, 30, 60, and 90 days of supplementation. In addition, on the same days, at 3 time points during the day (30 min before lunch/in the afternoon/30 min before dinner) subjects were asked to rate their fatigue level, and mental and physical performance using NRS. Sleep quality on the previous night (duration and variation from habitual duration) was assessed in the evening (before dinner) after 30, 60, and 90 days of supplementation.

Compliance was assessed via a specific questionnaire, which was completed by each participant on a daily basis. Subjects came to the study centers for the inclusion visit and baseline day, and questionnaires on any other day were completed at home. Compliance was high during the study, except for two major protocol violations: one subject stopped taking the supplement temporarily between D30 and D60 and one took the capsules at another time than morning.

### 2.5. Study Endpoints

The MFI includes 20 items that are each rated on a 5-point scale. From these 20 items are derived 5 subscales scores (expressed in arbitrary units (a.u.)) in the following domains: general fatigue, physical fatigue, mental fatigue, activity reduction and motivation reduction; higher scores indicate greater fatigue. The MFI questionnaire has been validated, both in English and Spanish languages [31,33] by successfully assessing, in different groups of population, internal consistency and convergent validity [31].

The primary efficacy variable was the general fatigue subscale score after 90 days of supplementation.

The secondary efficacy variables included the general fatigue subscale score after 1, 15, 30, and 60 days and the MFI subscale scores for physical fatigue, mental fatigue, reduced activity, and reduced motivation after 1, 15, 30, 60, and 90 days of supplementation. Other secondary criteria were assessed using Numerical Rating Scales (NRS), which are non-specific tools for the evaluation of fatigue and performance, but are nonetheless validated in other areas, such as pain [34]. NRS for fatigue, stress, mental, and physical performance at the same timepoints were additional secondary endpoints, as were the percentages of product satisfaction for each domain. NRS were on an 11-point scale. Subject satisfaction was measured using a 4-category rate questionnaire from not satisfied at all (score 1) to entirely satisfied (score 4) for physical fatigue, intellectual fatigue, stress level, and overall satisfaction domains.

### 2.6. Statistical Considerations

The sample size was calculated for the primary endpoint MFI-general fatigue at 90 days. A total of 73 subjects was needed to show a difference of 2.0 a.u., considered as the minimal clinically important difference [35], associated with a standard deviation of 6.0 a.u. for the primary endpoint, using a two-sided comparison with a type 1 error rate controlled at 5% and a power of 80%. The primary endpoint analysis was performed on the per-protocol population, defined as all included subjects who presented no major protocol deviation (*n* = 103). The primary analysis was the comparison of MFI at baseline vs. 90 days, using a mixed model for repeated measurements (MMRM), including time points (baseline, D1, D15, D30, D60, D90) as categorical effects and subject as a random effect. A Dunnett adjustment was performed for comparison between baseline and the other time points for controlling the type 1 error rate. For NRS, the same MMRM was used but including in addition the time of day (before lunch, afternoon, evening) and the interactions between time points and time of day. The same adjustment as for MFI for comparison of the other time point was used. MFI and NRS assessments were analyzed using a 5% 2-sided significance level. No formal statistical comparison was planned for satisfaction endpoints.

## 3. Results

### 3.1. Fatigue-Related and Energy-Related Outcomes

#### 3.1.1. MFI Scores

General fatigue subscale score, the primary study endpoint, was decreased by 7.55 points [95% CI −8.44; −6.66] (41.8%), compared with baseline (*p* < 0.0001) after 3 months of supplementation. During the same period, subscale scores for reported physical and mental fatigue decreased by 6.10 points [95% CI −6.95; −5.25] (35.8%) and 3.91 points [95%CI −4.45; −3.38] (26.1%), respectively. All the components of the MFI showed a significant decrease from D15 up to D90 following the first intake of the study product (Table 2).

#### 3.1.2. Self-Evaluation of Fatigue and Performance via Numerical Rating Scales

The self-estimated fatigue level recorded in the evening was significantly decreased, by 2.94 points [95% CI −3.53; −2.35] and the self-rated level of mental and physical performance was significantly increased, by 2.21 points [95% CI −1.58; −2.85] after a 3-month intake of the supplement. Statistically significant improvements were observed from 2 weeks after the initiation of the supplementation for all the assessed outcomes (Table 2). Fatigue and performance ratings before lunch and in the afternoon displayed the same trends, with significant favorable changes observed as soon as 2 weeks after the initiation of supplementation, which were sustained and strengthened up to 3 months (data not shown). Improvements were thus noticeable and consistent throughout the whole day.

#### 3.1.3. Satisfaction and Perceived Energy Scores

**Figure 1:**
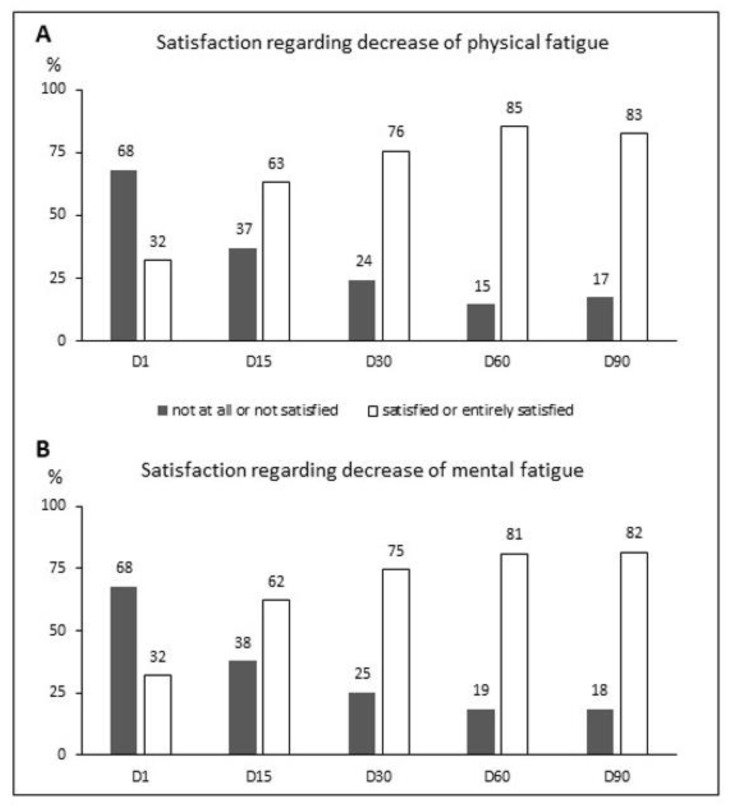
Percentage of population self-reporting the indicated level of satisfaction regarding the ability of the product to reduce physical fatigue (**A**) and mental fatigue (**B**), from D1 to D90 following supplementation. D—day.

Regarding satisfaction with perceived physical and mental fatigue (Figure 1), there was a clear trend towards a decreased number of non-satisfied participants over time. Overall, 68% of subjects were unsatisfied when they started supplementation and this percentage dropped down to 17% after 90 days (data not shown). More precisely, after 3 months 83% of individuals were satisfied with the decrease of physical fatigue and 82% were satisfied regarding the decrease of mental fatigue, whereas only 32% had been satisfied at study onset.

**Figure 2:**
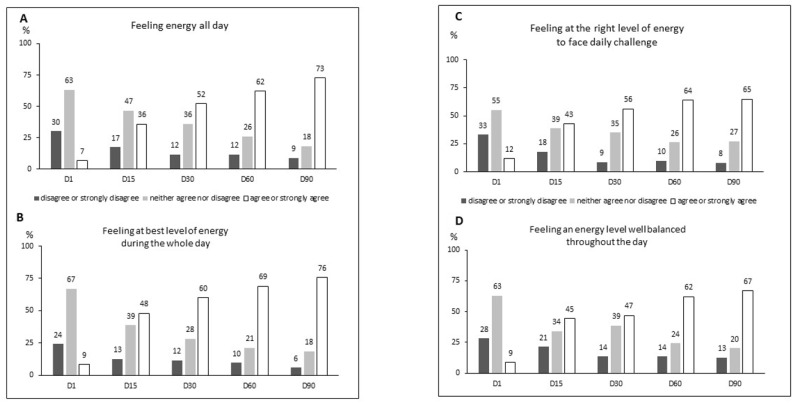
Percentage of population self-reporting the indicated level of agreement with proposed statements evaluating satisfaction vs. “feeling energy all day” (**A**), “feeling at best level of energy during the whole day” (**B**), “feeling at the right level of energy to face daily challenge” (**C**) or “feeling an energy level well balanced throughout the day” (**D**) from D1 to D90 following supplementation. D—day.

When the nature and extent of the feelings of energy were investigated among the subjects, very clear improvements were seen in terms of satisfaction, which steadily increased during the study (Figure 2). Only 7% of subjects agreed they had a “feeling of energy” at study onset, while 73% agreed with this statement after 3 months. Similarly, between the start and end of the study, subjects agreement that they were “feeling at their best level of energy during the whole day” increased from 9% up to 76% and positive responses to the question of whether they were feeling “the right level of energy” increased from 12% up to 65%. Most subjects (67%) agreed that their “energy level [was] balanced throughout the day” after 3 months of supplementation, vs. a minority of subjects (9%) at study onset. In addition (data not shown), 65% of individuals reported that they were ‘more focused on daily challenges’ after 3 months, in comparison with 34% after 2 weeks, 49% after 1 month and 54% after 2 months.

### 3.2. Satisfaction and Perceived Stress

After 3 months of supplementation, numerical ratings related to stress perception decreased by 2.30 points [95% CI −2.92; −1.68], relative to baseline ratings. Decreases in NRS for stress were significant from 2 weeks after onset of supplementation (*p* < 0.0001 at all times). In addition, 80% of the population was satisfied by the level of stress reduction they experienced after 3 months, vs. 31% at study onset (data not shown).

### 3.3. Duration of Sleep

After 3 months of supplementation, 79% of the subjects declared sleeping between 6 h and 8 h at night; a similar percentage (80% of the subjects) was observed after 1 month of supplementation. More than 60% of subjects observed that they did not notice any difference from their usual sleep time, only 14% of subjects reported they slept less and 12% felt they had slept more than usual on the previous night. Only 2% of subjects agreed they had a “feeling of energy through the day and good night sleep” at study onset, while 59% agreed with this statement after 3 months of supplementation.

No adverse events have been reported throughout the study.

## 4. Discussion

Daily intake of a food supplement containing a combination of vitamins, minerals and *Panax ginseng* root dry extract (G115^®^) significantly decreased the perception of fatigue compared to baseline in subjects who were suffering from non-disease-related persistent fatigue. This improvement was seen after two weeks and increased consistently with the treatment duration, up to the end of the study. In particular, the general fatigue subscale score of the MFI decreased by more than 7.55 points [95%CI −8.44; −6.66]. A decrease of 2.0 points is considered as the minimal clinically important difference on this scale [35]. Feelings of physical fatigue decreased by more than one-third, and those of mental fatigue decreased by more than one-quarter after three months. Perceived activity and motivation levels were significantly increased, gaining around 40%; these dimensions refer to the influence of physical and psychological factors on the level of activity and to the motivation needed for starting any activity. Their improvement confirmed by subjects’ own evaluations (NRS ratings of mental and physical performance) is thus a good indicator that subjects feel that their physical and mental energy is supported by the supplementation. Participants also self-reported enhanced satisfaction with the supplementation regarding decreased mental and physical fatigue, as well as improvements in the level and quality of feelings of energy.

Among the strengths of this study, one is the assessment of its primary criteria with the MFI questionnaire, which has been validated in various population groups, including healthy volunteers, individuals with chronic fatigue, and subjects with strenuous occupations [31,36]. The population in the current study exhibited mean general fatigue subscale scores that were higher than those of a healthy general population (e.g., 10.8 points in healthy Swedish women) [2] but not as high as those observed in chronic fatigue syndrome (e.g., 25.4 points in a group of 357 chronically fatigued patients) [31], and similar to those observed in medical students (16.1points) or soldiers while training (17.0 points) [31]. The findings of this study could thus be extended to populations experiencing everyday malaise (fatigue and reduced motivation), independently of a specific disease.

In addition, this study aimed at monitoring the self-perceptions of mental and physical fatigue in users of a food supplement, in real-world conditions. In particular, such data, obtained with a validated assessment tool, have been lacking for intermediate durations of intake (15 days, 1 or 2 months) while it is suspected that supplements are often taken for less than three months. A limitation of the study is its open-label design, which is however consistent with the objective of the study. It was not intended to provide a placebo-controlled demonstration of the benefits associated with the intake of *Panax ginseng* root extract G115^®^ vitamin and mineral combination, which has been addressed in other publications (for a recent review, see [19]).The improvements in self-perception and satisfaction levels started from the second week following initiation of the supplementation and were consistently enhanced with supplement duration, up to the end of the study period, and this adds to the body of evidence that the benefit experienced by the subjects is real compared to initial state; one might estimate that a simple placebo effect would not have lasted for such a long time (three months), with such a level of magnitude and in such a sustained manner (with the proportion of satisfied participants continuing to increase up to the end of the study).

## 5. Conclusions

Overall, this study provides new knowledge about the perception and self-evaluation of fatigue and feelings of energy, using the validated MFI questionnaire, in adults who recovered from fatigue associated with modern lifestyles, thanks to the daily intake of a vitamin and mineral supplement containing G115^®^ extract of the root of *Panax ginseng*. Whilst this was not a placebo-controlled trial, the data from this study suggests both that a full randomized controlled trial is warranted and that the endpoints identified above would be suitable measures.

## Figures and Tables

**Table 1:** Baseline characteristics of subjects (*n* = 103).

Demographic Characteristics	
Age (years; mean ± SD)	35.1 ± 7.87
Gender (% male)	25.2
Professional activity	
Full time remunerated (%)	62.1
Part time remunerated; others (%)	24.3
Students	13.6
**Lifestyle Characteristics**	
Smoking habits (% non-smokers)	98.1
Alcohol habits (% with no intake)	81.6
Energy drinks consumption (% non-consumers)	92.2
Snack intake (% consumers once or more weekly)	50.5
Physical Activity (% with no medium or enhanced weekly sporting activities)	89.3
**MFI-20 Subscale Scores (arbitrary units, mean** **± SD** **)**	
General fatigue	17.9 ± 2.04
Physical fatigue	16.7 ± 2.57
Mental fatigue	14.6 ± 1.71
Reduced activity	14.1 ± 4.16
Reduced motivation	13.5 ± 3.36
**Numerical Rating Scores (11-point scale)**	
Fatigue	8.1 ± 1.76
Mental & physical performance	3.8 ± 2.60
Stress	6.3 ± 2.52

MFI, Multidimensional Fatigue Inventory; SD, standard deviation.

**Table 2:** Mean differences (expressed in arbitrary units) between baseline and post baseline visit of MFI subscale scores and numerical rating scores for fatigue, performance, and stress at different times of supplementation with a vitamin and mineral supplement containing *Panax ginseng* extract (G115^®^) (*n* = 103).

	D1	D15	D30	D60	D90
**MFI-20 subscale scores**					
General fatigue	−0.51 [−1.40; 1.39]	−3.97 [−4.86;−3.08] **	−5.64 [−6.53;−4.75] **	−6.97 [−7.86;−6.08] **	−7.55 [−8.44;−6.66] **
Physical fatigue	−0.21 [−0.64; 1.06]	−2.40 [−3.25;−1.55] **	−3.70 [−4.55;−2.85] **	−4.93 [−5.78;−4.08] **	−6.10 [−6.95;−5.25] **
Mental fatigue	−0.44 [−0.97; 0.10]	−2.09 [−2.62; −1.55] **	−2.93 [−3.47; −2.40] **	−3.53 [−4.07; −3.00] **	−3.91 [−4.45; −3.38] **
Reduced activity	−0.52 [−1.42; 0.37]	−2.29 [−3.18; −1.40] **	−3.73 [−4.62; −2.84] **	−4.75 [−5.64; −3.85] **	−5.44 [−6.33; −4.54] **
Reduced motivation	−0.32 [−1.09; 0.45]	−2.82 [−3.58; −2.05] **	−3.74 [−4.51; −2.97] **	−4.68 [−5.45; −3.91] **	−5.72 [−6.49; −4.95] **
**Assessments by NRS**					
Fatigue	−0.84 [−1.43; −0.26] *	−2.20 [−2.79; −1.62] **	−2.66 [−3.25; −2.07] **	−3.04 [−3.63; −2.45] *	−2.94 [−3.53; −2.35] **
Mental & physical performance	0.58 [−0.05; 1.22]	1.45 [0.81; 2.08] **	2.16 [1.52; 2.79] **	2.20 [1.57; 2.84] **	2.21 [1.58; 2.85] **
Stress	−0.58 [−1.20; 0.04]	−1.62 [−2.24; −1.00] **	−1.93 [−2.55; −1.31] **	−2.48 [−3.10; −1.85] **	−2.30 [−2.92; −1.68] **

Least squares mean difference [95% confidence interval]: *: *p* = 0.0015; **: *p* < 0.0001. All NRS values are those reported in the evening. D—day; MFI—Multidimensional Fatigue Inventory; NRS—Numerical Rating Scores.

## Data Availability

Qualified researchers may request access to patient-level data and related documents [including, e.g., the clinical study report, study protocol with any amendments, blank case report form, statistical analysis plan, and dataset specifications]. Patient-level data will be anonymized, and study documents will be redacted to protect the privacy of trial participants. Further details on Sanofi’s data sharing criteria, eligible studies, and process for requesting access can be found at https://www.clinicalstudydatarequest.com (last accessed 8 June 2021).
